# Predictors of Loss of Ambulation in Duchenne Muscular Dystrophy: A Systematic Review and Meta-Analysis

**DOI:** 10.3233/JND-230220

**Published:** 2024-04-30

**Authors:** E. Landfeldt, A. Alemán, S. Abner, R. Zhang, C. Werner, I. Tomazos, N. Ferizovic, H. Lochmüller, J. Kirschner

**Affiliations:** a IQVIA, Stockholm, Sweden; b Department of Pediatrics, Division of Neurology, Children’s Hospital of Eastern Ontario, Research Institute, University of Ottawa, Ottawa, ON, Canada; c IQVIA, London, UK; d PTC Therapeutics Sweden AB, Askim, Sweden; e PTC Therapeutics Germany GmbH, Frankfurt, Germany; f PTC Therapeutics Inc, South Plainfield, NJ, USA; g Department of Neuropediatrics and Muscle Disorders, Medical Center, University of Freiburg, Faculty of Medicine, Freiburg, Germany; h Department of Medicine, Division of Neurology, The Ottawa Hospital, Brain and Mind Research Institute, University of Ottawa, Ottawa, Canada

**Keywords:** Motor function, 6MWT, Neuromuscular Disease, treatment, guidelines, NOS

## Abstract

**Objective:**

The objective of this study was to describe predictors of loss of ambulation in Duchenne muscular dystrophy (DMD).

**Methods:**

This systematic review and meta-analysis included searches of MEDLINE ALL, Embase, and the Cochrane Database of Systematic Reviews from January 1, 2000, to December 31, 2022, for predictors of loss of ambulation in DMD. Search terms included “Duchenne muscular dystrophy” as a Medical Subject Heading or free text term, in combination with variations of the term “predictor”. Risk of bias was assessed using the Newcastle–Ottawa Scale. We performed meta-analysis pooling of hazard ratios of the effects of glucocorticoids (vs. no glucocorticoid therapy) by fitting a common-effect inverse-variance model.

**Results:**

The bibliographic searches resulted in the inclusion of 45 studies of children and adults with DMD from 17 countries across Europe, Asia, and North America. Glucocorticoid therapy was associated with delayed loss of ambulation (overall meta-analysis HR deflazacort/prednisone/prednisolone: 0.44 [95% CI: 0.40–0.48]) (*n* = 25 studies). Earlier onset of first signs or symptoms, earlier loss of developmental milestones, lower baseline 6MWT (i.e.,<350 vs. ≥350 metres and <330 vs. ≥330 metres), and lower baseline NSAA were associated with earlier loss of ambulation (*n* = 5 studies). Deletion of exons 3–7, proximal mutations (upstream intron 44), single exon 45 deletions, and mutations amenable of skipping exon 8, exon 44, and exon 53, were associated with prolonged ambulation; distal mutations (intron 44 and downstream), deletion of exons 49–50, and mutations amenable of skipping exon 45, and exon 51 were associated with earlier loss of ambulation (*n* = 13 studies). Specific single-nucleotide polymorphisms in *CD40* gene rs1883832, *LTBP4* gene rs10880, *SPP1* gene rs2835709 and rs11730582, and *TCTEX1D1* gene rs1060575 (*n* = 7 studies), as well as race/ethnicity and level of family/patient deprivation (*n* = 3 studies), were associated with loss of ambulation. Treatment with ataluren (*n* = 2 studies) and eteplirsen (*n* = 3 studies) were associated with prolonged ambulation. Magnetic resonance biomarkers (MRI and MRS) were identified as significant predictors of loss of ambulation (*n* = 6 studies). In total, 33% of studies exhibited some risk of bias.

**Conclusion:**

Our synthesis of predictors of loss of ambulation in DMD contributes to the understanding the natural history of disease and informs the design of new trials of novel therapies targeting this heavily burdened patient population.

## INTRODUCTION

Duchenne muscular dystrophy (DMD) is a severe, childhood-onset muscular dystrophy characterized by progressive muscle weakness starting during early childhood and typically leading to loss of independent ambulation during early adolescence [1]. As the disease progresses, respiratory insufficiency and dilatative cardiomyopathy increasingly contribute to the clinical presentation and finally lead to high mortality during young adulthood [2]. DMD is caused by mutations of the dystrophin gene on the X-chromosome and therefore mainly affects boys. About two thirds of patients have larger mutations with deletion or duplication of several exons, while the remaining patients harbor point mutations including premature stop codon (nonsense) and splice site mutations [3]. While DMD is typically associated with almost complete loss of dystrophin expression, Becker muscular dystrophy (BMD) is an allelic disorder caused by hypomorphic mutations associated with some residual dystrophin expression and a milder phenotype.

Loss of ambulation represents a critical disease milestone in the progression of DMD that can have a significant impact on the physical, social, and emotional well-being of patients and their families. Indeed, from a clinical viewpoint, losing the ability to walk independently typically marks the start of a more forceful disease trajectory, including increasingly pronounced upper extremity weakness and compromised cardiac and respiratory functioning [4]. Moreover, from the perspective of the affected patient, becoming non-ambulatory also has a detrimental impact on personal autonomy and independence, including the ability to perform activities of daily living and fully take part in recreational and social events, in particular outside of the home. For these reasons, losing independent ambulation can be emotionally difficult and distressing, resulting in feelings of helplessness, sadness, frustration, anger, and fear about the future [5, 6]. Additionally, as patients require further help and support, loss of ambulation would be expected to have a non-trivial impact also on the well-being of informal caregivers, such as parents and friends [7, 8].

Studies have shown that age at loss of ambulation in DMD varies substantially between patients and cohorts. However, despite its relevance to patients, clinicians, and researchers, we presently lack an up-to-date evidence review of factors influencing this milestone in DMD. The objective of this systematic literature review was to describe the published evidence of predictors of loss of ambulation in patients with DMD, with data of the effects of glucocorticoids synthesized in a meta-analysis.

## MATERIAL AND METHODS

### Search Strategy and Selection Criteria

We performed a systematic literature review in accordance with the Preferred Reporting Items for Systematic Reviews and Meta-Analyses (PRISMA) statement [9]. We searched MEDLINE ALL, Embase, and the Cochrane Database of Systematic Reviews for studies reporting evidence of predictors of loss of ambulation in patients with DMD published between January 1, 2000 (to ensure relevance to current care practices) and December 31, 2022. We used the search terms “Duchenne muscular dystrophy” as a Medical Subject Heading or free text term, in combination with variations of the term “predictor” (full search strings are provided in the supplemental material online).

To be considered eligible for inclusion, studies were required to report evidence of a predictor of loss of ambulation, defined as any factor –either endogenous (e.g., *DMD* mutations or genetic modifiers) or exogenous (e.g., pharmacological interventions, including exposure to glucocorticoids) –significantly associated with the timing of loss of ambulation. We considered studies of male patients with DMD of any age exposed to any treatments. We included studies of any type, reported in any language. We did not include editorial letters or conference abstracts (as they lack details essential for meaningful synthesis) and did not formally include identified systematic reviews (but screened their reference lists for potential publications).

### Screening and Data Extraction

After initial screening of publication titles, articles were assessed for eligibility by EL and SA. The following information was subsequently extracted from all articles that met the review inclusion criteria: Author; title; study year; geographical setting(s); study design; site(s)/data source(s); study period; sample population characteristics; case ascertainment; pharmacological interventions (incl. number of exposed, dose, and duration of exposure); method of analysis; and outcome results. Upon identification of the relevant literature, two investigators (EL and SA) systematically screened reference lists of all included publications with the aim to identify additional records of interest not captured by the search strategy.

### Risk of Bias

Risk of bias of included records were assessed by two investigators (EL and AA) using the Newcastle-Ottawa Scale (NOS) [10]. The NOS assesses risk of bias of non-randomized research in three dimensions: (1) the selection of the study groups; (2) the comparability of the groups; and (3) the ascertainment of either the exposure or outcome of interest for case-control or cohort studies, respectively. For each category, a score rating is assigned based on the NOS criteria (maximum score: ◊◊◊◊ for selection, ◊◊ for comparability, and ◊◊◊ for outcome) [10]. To ascertain selection, we required patients to be diagnosed with DMD (score: ◊), that the diagnosis was established via genetic testing and/or muscle biopsy (score: ◊), and that the sample was not restricted in terms of DMD mutation type or other markers limiting representativeness (score: ◊) [assessment of the non-exposed cohort was not applicable, and all studies were thus assigned a score (◊) for this criterion]; to ascertain comparability, we required details of the number of patients and exposure to glucocorticoids in the sample population (score for two details: ◊◊; score for at least one detail: ◊); and to ascertain outcome, we required that information regarding loss of ambulation was extracted from clinical charts or registries/databases containing physician-reported or administrative data [e.g., governmental population-based registries or claims databases] (score: ◊), a minimal follow-up of five years for prospective studies [given the frequency of loss of ambulation] (score: ◊), and that less than 25% of the total sample were lost to follow-up during the study period (score: ◊). Studies were considered to be characterized by risk of bias if not attributed the maximum score in all categories of the NOS.

### Statistical Analysis

We performed meta-analysis pooling of hazard ratios of the effects of glucocorticoids (vs. no glucocorticoid therapy) by fitting a common-effect inverse-variance model using the *metan* Stata module (Stata 15, StataCorp, College Station, TX, USA) [11]. Previous research (e.g., [12, 13]) has shown that the effect of glucocorticoids may vary across available agents. We therefore conducted the meta-analysis separately for three mutually exclusive groups defined in terms of glucocorticoid agent/agents (as reported in the included publications): (1) deflazacort, prednisone, and/or prednisolone, (2) prednisone and/or prednisolone, and (3) deflazacort. We ran the analysis with estimates from all eligible studies, as well as from the subset of records that did not exhibit any risk of bias (as assessed using the NOS). Heterogeneity was assessed using the I^2^ index [14].

## RESULTS

**Fig. 1:**
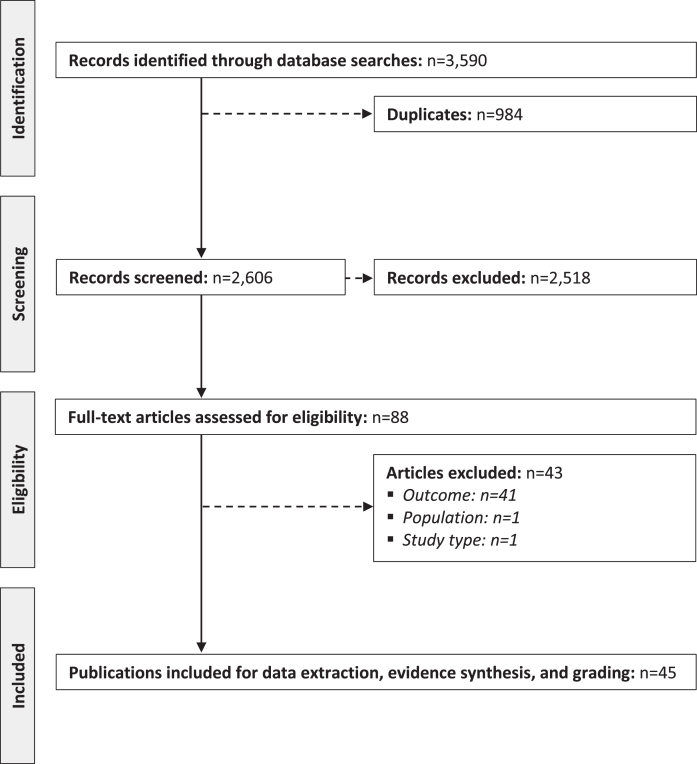
PRISMA diagram of the selection process of the included publications.

The bibliographic searches resulted in the identification of 3,590 publications, of which 45 [12, 13, 15–57] were included for extraction and synthesis (Fig. 1). All except one study [57] were observational in nature. In total, included publications involved patients from a total of 17 countries (i.e., Bulgaria, Canada, China, the Czech Republic, Denmark, France, Germany, Hungary, Italy, Japan, the Netherlands, Poland, Serbia, Switzerland, Turkey, the United Kingdom [UK], and the United States of America [USA]) (Table 1). However, 24% (11 of 45) were based on data from unspecified multi-national cohorts [12, 16–18, 30, 33, 34, 37, 38, 43, 57].

**Table 1:** Characteristics of included studies

Author (year) [country]	Study design	Site(s)/data source(s)	Study period	Sample, n (age)	Case ascertainment	Pharmacological intervention(s)	n (%) exposed	Dose, mean	Duration of exposure, mean (SD)
Barber et al. (2013) [US] [15]	Retrospective cohort study	MD STAR*net* (US, multi-centre)	1982–2010	462 patients with DMD (mean age: NR, range: NR)	NR^a^	Glucocorticoids (DFZ, PDN, or PRED)	291 (63%)	NR	4.1 (3.4) years
Barnard et al. (2020) [US] [52]	Prospective cohort study	ImagingDMD (US, multi-centre)	2010–NR	160 patients with DMD (mean age: 8 years, range: 4–18 years)	NR	Glucocorticoids (DFZ, PDN, or PRED)	118 (74%)	NR	NR
						Ataluren	19 (12%)	NR	NR
						Eteplirsen	15 (9%)	NR	NR
Bello et al. (2015) [*] [12]	Prospective cohort study	CINRG DNHS (multi-country, multi-centre)	NR–2013	340 patients with DMD (mean age: 14 years, range: 2–28 years)	NR^a^	Glucocorticoids (DFZ, PDN, or PRED)	277 (81%)	Regimen-specific (see article for details)	4.0 years (3.3) (range: 0.1–18.3) years
Bello et al. (2015) [*] [16]	Prospective cohort study	CINRG DNHS (multi-country, multi-centre)	NR	340 patients with DMD (mean age: NR, range: 2–28 years)	NR^a^	Glucocorticoids (agents NR)	252 (74%)	NR	>1 year (before loss of ambulation)
Bello et al. (2016) [*] [17]	Prospective cohort study	CINRG DNHS (multi-country, multi-centre)	2006–2009	212 patients with DMD (mean age: NR, range: NR)	NR^a^	Glucocorticoids (DFZ, PDN, or PRED)	157 (74%)	NR	>1 year (before loss of ambulation)
Bello et al. (2016) [*] [18]	Prospective cohort study	CINRG DNHS (multi-country, multi-centre)	NR	109 patients with DMD (mean age: NR, range: NR)	NR^a^	Glucocorticoids (agents NR)	NR	NR	>1 year (before loss of ambulation)
Biggar et al. (2001) [CA] [19]	Retrospective cohort study	The Bloorview MacMillan Children’s Center (Toronto, CA)	1993–1999	54 patients with DMD (mean age: 12 years, range: 7–15 years)	Age at onset of symptoms (<5 years of age), male sex, proximal muscle weakness, increased serum creatine kinase levels, and muscle biopsy and/or genetic testing	Glucocorticoids (DFZ)	30 (56%)	*Initial dose* •0.9 mg/kg/day *At 10 years of age* • 0.76 (0.19) mg/kg/day *At 15 years of age* • 0.61 (0.20) mg/kg/day	3.2 (1.3) years
Bonifati et al. (2006) [IT] [20]	Retrospective cohort study	Database (name and location NR)	NR	48 patients with DMD (mean age: 8 years, range: 4–12 years)	Genetic testing	Glucocorticoids (DFZ or PDN)	48 (100%)	*First year of treatment* •DFZ: 0.9 mg/kg/day •PDN: 0.75 mg/kg/day *Second year of treatment (until loss of ambulation)* •DFZ: 1.5 mg/kg every other day •PDN: 1.8 mg/kg/day every other day	NR
Chen et al. (2020) [CN] [21]	Retrospective cohort study	The First Affiliated Hospital of Sun Yat-sen University (Guangzhou, CN)	NR	326 patients with DMD (mean age: 8 years, range: 4–12 years)	Genetic testing	Glucocorticoids (DFZ or PDN)	144 (44%)	NR	NR
Ciafaloni et al. (2016) [US] [22]	Retrospective cohort study	MD STAR*net* (US, multi-centre)	1982–2009	825 patients with DMD (mean age: NR, range: NR)	NR^a^	Glucocorticoids (agents NR)	808 (49%)	NR	NR
Fischmann et al. (2012) [CH] [53]	Prospective cohort study	NR	NR	20 patients with DMD (mean age: 11 years, range: 5–23 years)	Genetic testing	NR	NR	NR	NR
Flanigan et al. (2013) [US] [23]	Retrospective cohort study	The United Dystrophinopathy Cohort (US, multi-centre)	NR	239 patients with DMD (mean age: NR, range: NR)	Age of onset of symptoms, disease progression characteristics, and genetic testing	Glucocorticoids (agents NA)	137 (57%)	NR	NR
Godi et al. (2016) [IT] [54]	Prospective cohort study	Neuromuscular centre (name and location NR, IT)	2009–NR	26 patients with DMD (median age: 9 years, range: 5–12 years)	Clinical history of early onset, progressive muscle weakness, increased serum creatine kinase levels, muscle biopsy and genetic testing	Glucocorticoids (agents NR)	25 (96%)	NR	NR
Haber et al. (2021) [US] [24]	Retrospective cohort study	MD STAR*net* (US, multi-centre)	1982–2012	358 patients with DMD (mean age: NR, range: NR)	NR^a^	Glucocorticoids (DFZ or PDN)	226 (63%)	NR	3.10 (SD: 2.84) years
Houde et al. (2008) [CA] [25]	Retrospective cohort study	The multidisciplinary Neuromuscular Clinic of the Marie-Enfant Rehabilitation Centre (Montreal, CA)	NR	79 patients with DMD (mean age: 11 years; range: NR)	Muscle biopsy and/or genetic testing	Glucocorticoids (DFZ)	37 (47%)	*Initial dose* •0.9 mg/kg per day (adjusted according to evolution or side effects to a maximum of 1 mg/kg) *At last visit* •0.69 (SD: 0.20) mg/kg	66 months
ACE inhibitors (agents NR)	30 (38%)	NR	NR
Hufton et al. (2017) [UK] [26]	Retrospective cohort study	Neuromuscular clinic (name and location NR, UK)	2005–2014	69 patients with DMD (mean age: 10 years; range: 5–18 years)	Genetic testing	Glucocorticoids (agents NA)	57 (83%)	NR	NR
Humbertclaude et al. (2012) [FR] [27]	Retrospective cohort study	The French dystrophinopathy database (UMD–DMD France) (FR, multi-centre)	NR	278 patients with DMD (mean age: 11 years; range: NR)	Genetic testing and loss of ambulation before 13 years of age	Glucocorticoids (agents NA)	0 (0%)	NA	NA
Kim et al. (2015) [US] [28]	Retrospective cohort study	MD STAR*net* (US, multi-centre)	1982–2011	477 patients with DMD (mean age: 7 years; range: NR)	NR^a^	Glucocorticoids (DFZ and/or PDN)	220 (46%)	NR	3.4 years
King et al. (2007) [US] [29]	Retrospective cohort study	The Ohio State University (Columbus, US)	2000–2003	143 patients with DMD (mean age: 16 years; range: 1–40 years)	Clinical presentation and genetic testing	Glucocorticoids (DFZ and/or PDN)	75 (52%)	•DFZ: 0.9 mg/kg/day •PDN: 0.75 mg/kg/day	8.04 (5.2) (range: 0.5–18.5) years
Koeks et al. (2017) [*] [30]	Retrospective cohort study	The TREAT-NMD global DMD database (multi-national, multi-centre)	2007–2013	5,345 patients with DMD (mean age: NR, range: NR)	Genetic testing	Glucocorticoids (DFZ, PDN, or PRED)	*Current use* •2,658 (50%) *Past use* •522 (10%)	NR	NR
Kosac et al. (2022) [RS] [31]	Retrospective cohort study	•Clinic for Neurology and Psychiatry for Children and Youth (Belgrade, RS) •Mother and Child Health Care Institute of Serbia “Dr Vukan Cupic” (Belgrade, RS)	NR	95 patients with DMD (mean age: 16 years, range: NR)	Genetic testing	Glucocorticoids (DFZ or PRED)	73 (77%)	NR	>1 year
Mazzone et al. (2013) [IT] [32]	Retrospective cohort study	IT, multi-centre	2008–2009	113 patients with DMD (mean age: 8 years, range: 4–17 years)	Genetic testing	Glucocorticoids (DFZ or PRED)	*Intermittent glucocorticoids* •40 (35%) *Daily glucocorticoids* •67 (59%)	*Intermittent regimen* •DFZ: 0.9 mg/kg/day •PDN: 0.75 mg/kg/day *Daily regimen:* •DFZ: 0.9 mg/kg/day •PDN: 0.75 mg/kg/day	NR
McDonald et al. (2018) [*] [33]	Prospective cohort study	CINRG DNHS (multi-country, multi-centre)	2006–2009; 2012–2016	403 patients with DMD (mean: 11 years, range: 2–28 years)	Clinical and molecular diagnostic picture consistent with typical DMD (see article for details)	Glucocorticoids (DFZ, PDN, and/or PRED)	330 (82%)	NR	Regimen-specific (see article for details)
McDonald et al. (2022) [*] [34]	Indirect treatment comparison study	•Study 019 (open-label study; multi-country, multi-centre [NCT01557400]) •CINRG DNHS (prospective cohort study; multi-country, multi-centre [NCT00468832])	•Study 019: NR •CINRG DNHS: 2006–2016	120 patients with DMD (distribution of age NR)	•Study 019: NR^a^ •CINRG DNHS: NR^a^	Ataluren	60 (50%)	40 mg/kg/day	NR^a^
Mendell et al. (2016) [US] [35]	Indirect treatment comparison study	•Study 201 (RCT; US; single-centre [NCT01396239]) •Study 202 (open label, multiple dose extension study; US, multi-centre)	NR	25 patients with DMD (mean age: 9 years, range: 7–12 years)	Genetic testing	Eteplirsen	12 (48%)	30mg/kg or 50mg/kg	≥3 years
Glucocorticoids (DFZ, PDN, or PRED)	25 (100%)	NR	≥24 weeks
Mendell et al. (2021) [US] [36]	Indirect treatment comparison study	•Study 201 (RCT; US; single-centre [NCT01396239]) •Study 202 (open label, multiple dose extension study; US, multi-centre [NCT01540409]) •The Italian Telethon and Leuven registries (multi-country, multi-centre)	NR	23 patients with DMD (mean age: 9 years, range: 7–12 years)	NR^a^	Eteplirsen	12 (52%)	•30mg/kg or 50mg/kg	≥4 years
Glucocorticoids (DFZ and/or PDN)	25 (100%)	•DFZ: 0.9 mg/kg/day •PDN: 0.75 mg/kg/day	NR
Mercuri et al. (2020) [*] [37]	Indirect treatment comparison study	•The STRIDE Registry (multi-country, multi-centre) •CINRG DNHS (multi-country, multi-centre)	•The STRIDE Registry: 2006–2018 •CINRG DNHS: NR^a^	362 patients with DMD (mean age: 11 years, range: 2–27 years)	NR^a^	Ataluren	181 (50%)	NR^a^	632 (363) (range: 5–1,453) days
						Glucocorticoids (agents NR)	NR	NR	•The STRIDE Registry: 995 (1,184) days •CINRG DNHS: 978 (1,166) days
Mitelman et al. (2022) [*] [38]	Indirect treatment comparison study	•Study 201 (RCT; US; single-centre [NCT01396239]) •Study 202 (open label, multiple dose extension study; US, multi-centre •Study 405 (retrospective cohort study; US, multi-centre) •CINRG DNHS (prospective cohort study; multi-country, multi-centre [NCT00468832])	NR	83 patients with DMD (mean age: 8 years, range 5–15 years)	•Study 201/202: NR^a^ •Study 405: NR^a^ •CINRG DNHS: NR^a^	Eteplirsen	12 (14%)	30 or 40 mg/kg/week	5.72 (SD: 0.90) (range: 4.13–6.88)
						Glucocorticoids (DFZ or PDN)	27 (100%)	NR	NR
Naarding et al. (2020) [NL] [55]	Prospective cohort study	Leiden University Medical Center (LUMC) (Leiden, the Netherlands)^b^	2013–2016	LUMC: 22 patients with DMD (median age: 9 years, IQR: 7–12 years)	Genetic testing	Glucocorticoids (DFZ or PDN)	LUMC: 18 (82%)	NR	NR
Pane et al. (2014) [IT] [39]	Prospective cohort study	IT, multi-centre	2008–2013	96 patients with DMD (mean age: 8 years, range: 5–13 years)	Genetic testing	Glucocorticoids (DFZ or PDN)	91 (95%)	Regimen-specific (see article for details)	NR
Rooney et al. (2020) [US] [56]	Prospective cohort study	ImagingDMD (US, multi-centre)	2011–2018	104 patients with DMD (median age: 9 years, range: 4–17 years)	NR^a^	Glucocorticoids (DFZ, PDN, or PRED)	90 (87%)	NR	NR
Schara et al. (2001) [DE] [40]	Retrospective cohort study	NR	NR	26 patients with DMD (mean age: NR, range: NR)	NR	Glucocorticoids (DFZ)	13 (50%)	•0.9 mg/kg/day	NR
Servais et al. (2015) [FR] [41]	Case series	•Pre-U7 (multi-country, multi-centre [NCT01385917]) •ULENAP (FR, multi-centre [NCT00993161])	NR^a^	35 patients with DMD (mean age: 14 years, range: 9–19 years)	NR^a^	Glucocorticoids (agents NR)	6 (17%)	•20 mg/day	NR
						ACI inhibitors (agents NR)	24 (69%)	NR	NR
Sherlock et al. (2022) [*] [57]	RCT	RCT (multi-country, multi-centre) (NCT02310763)	NR^a^	120 patients with DMD (mean age: 9 years, range NR)	NR^a^	Glucocorticoids (agents NR)	120 (100%)	NR	≥6 months
Silversides et al. (2003) [CA] [42]	Retrospective cohort study	The Bloorview MacMillan Children’s Center (Toronto, CA)	1998–2002	33 patients with DMD (mean age: 15 years, range: 10–18 years)	Age at onset of symptoms (<5 years of age), male sex, proximal muscle weakness, increased serum creatine kinase levels, and muscle biopsy and/or genetic testing	Glucocorticoids (DFZ)	21 (64%)	*Initial dose* •0.9 mg/kg/day *At 10 years of age* •0.76 (0.19) mg/kg/day *At 15 years of age* •0.61 (0.20) mg/kg/day *At 18 years of age* •0.59 (0.15) mg/kg/day	5.1 (SD: 2.4) years
						ACE inhibitors (agents NR)	6 (18%)	NR	NR
						Cardiotonic agents (digoxin)	3 (9%)	NR	NR
Spitali et al. (2020) [*] [43]	Retrospective cohort study	•Undisclosed cohort (multi-country, multi-centre) •The Bio-NMD cohort (multi-country, multi-centre) •CINRG DNHS (prospective cohort study; multi-country, multi-centre [NCT00468832])	NR	437 patients with DMD (mean: NR, range: NR)	NR^a^	Glucocorticoids (agents NR)	NR	NR	≥1 year
Takeuchi et al. (2013) [JP] [44]	Retrospective cohort study	The Remudy database (JP, multi-centre)	2009–2012	553 patients with DMD (mean age: 15 years, range: NR)	Genetic testing	Glucocorticoids (PRED)	242 (44%)	NR	NR
van den Bergen et al. (2014) [NL] [46]	Retrospective cohort study	The Dutch Dystrophinopathy Database (DDD) (NL, multi-centre)	1961–1974; 1980–2006	336 patients with DMD (mean age: 15 years, range: NR)	Male sex, genetic testing and/or muscle biopsy, and loss of ambulation before 13 years of age	Glucocorticoids (agents NR)	165 (49%)	NR	NR
van den Bergen et al. (2014) [NL] [45]	Retrospective cohort study	The Dutch Dystrophinopathy Database (DDD) (NL, multi-centre)	NR	114 patients with DMD (mean age: NR, range: NR)	Genetic testing	Glucocorticoids (agents NR)	48 (42%)	NR	NR
van den Bergen et al. (2015) [†] [47]	Retrospective cohort study	Neuromuscular databases (multi-country, multi-centre)	NR	336 patients with DMD (mean age: NR, range: NR)	Muscle biopsy and/or genetic testing	Glucocorticoids (agents NR)	102 (30%)	NR	NR
Vry et al. (2016) [‡] [48]	Retrospective cohort study	The TREAT-NMD global DMD database (multi-national, multi-centre)	2011–2012	1,062 patients with DMD (mean age: 13 years, range: 1–46 years)	Database-specific (see article for details)	Glucocorticoids (agents NR)	792 (75%)^c^	NR	NR
Wang et al. (2014) [US] [50]	Retrospective cohort study	DuchenneConnect (now the Duchenne Registry) (US, multi-centre)	2007–2011	1,057 patients with DMD (mean age: NR, range: NR)	NR	Glucocorticoids (DFZ and/or PDN)	633 (60%)	NR	NR
Wang et al. (2018) [US] [49]	Retrospective cohort study	DuchenneConnect (now the Duchenne Registry) (US, multi-centre)	NR	765 patients with DMD (mean age: NR, range: NR)	NR	Glucocorticoids (DFZ and/or PDN)	NR	NR	NR
Yılmaz et al. (2004) [TR] [51]	Retrospective cohort study	NR	NR	88 patients with DMD (mean age: 7 years, range: 3–13 years)	NR	Glucocorticoids (PRED)	66 (75%)	0.75 mg/kg/day	2.75 (1.1) (range: 1.5–5) years
Zhang et al. (2021) [CN] [13]	Retrospective cohort study	Neuromuscular centres (see article for details) (CN, multi-centre) part of TREAT-NMD	2015–2019	967 patients with DMD (mean age: NR, range: NR)	Age at onset of symptoms (<5 years of age), elevated serum creatine kinase (CK) levels, and genetic testing	Glucocorticoids (DFZ, PDN, and/or PRED)	530 (55%)	•DFZ: 0.9 mg/kg/day •PDN/PRED: 0.3–0.75 mg/kg/day	NR

Note: Canada (CA). China (CN). Deflazacort (DFZ). Duchenne muscular dystrophy (DMD). France (FR). Germany (DE). Italy (IT). Japan (JP). Not applicable (NA). Not reported (NR). Prednisolone (PRED). Prednisone (PDN). Randomized controlled trial (RCT). Serbia (RS). The Netherlands (NL). Turkey (TR). United Kingdom (UK). United States of America (US). *Multi-national (see article for details). †The Netherlands, Italy, France, and the United Kingdom. ‡Bulgaria, the Czech Republic, Denmark, Germany, Hungary, Poland, and the United Kingdom. ^a^Details not reported but provided in referenced publications. ^b^Results for the Cincinnati Children’s Hospital Medical Center (CCHMC) cohort were not significant. ^c^Past and current use.

### Predictors of loss of ambulation in DMD

#### Glucocorticoid exposure

**Fig. 2:**
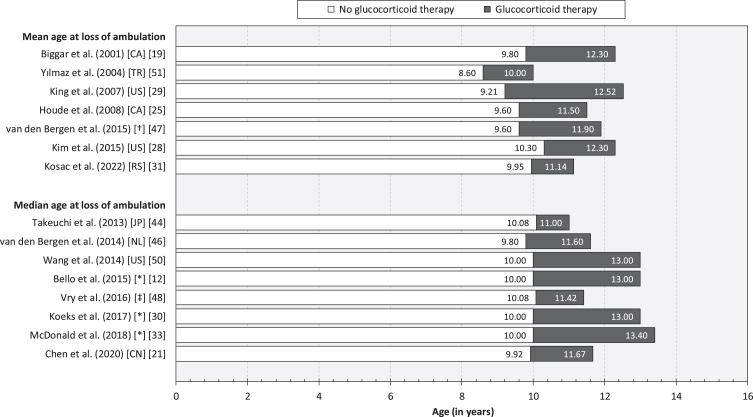
Mean and median age at loss of ambulation, by glucocorticoid therapy. Note: Canada (CA). China (CN). Japan (JP). Serbia (RS). The Netherlands (NL). Turkey (TR). United States of America (US). * Multi-national (see article for details). †The Netherlands, Italy, France, and the United Kingdom. ‡Bulgaria, the Czech Republic, Denmark, Germany, Hungary, Poland, and the United Kingdom.

**Fig. 3:**
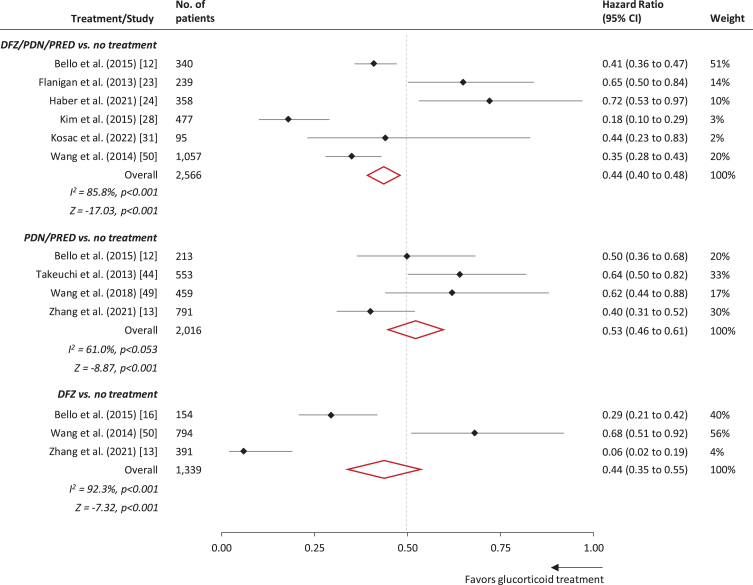
Forest plot of treatment effect of glucocorticoids on loss of ambulation in patients with DMD. Note: Estimates from Bello et al. [16], Bello et al. [17], and Wang et al. [48] were excluded from the meta-analysis as their respective patient cohorts were represented in other included studies of larger sample size (in some cases within treatment strata). Confidence interval (CI). Deflazacort (DFZ). Prednisolone (PRED). Prednisone (PDN).

We identified 25 observational studies reporting evidence of benefits of glucocorticoids on loss of ambulation in patients with DMD [12, 13, 15–21, 23–25, 28–31, 33, 40, 42, 44, 46, 48–51] (Table 2). Estimates of the mean and median age at loss of ambulation are presented in Fig. 2. A forest plot of hazard ratios associated with glucocorticoid therapy (vs. no glucocorticoid therapy) are presented in Fig. 3. The overall hazard ratio (HR) was estimated at 0.44 (95% CI: 0.40–0.48) for glucocorticoid therapy (deflazacort/prednisone/prednisolone), 0.53 (0.46–0.61) for prednisone/prednisolone therapy, and 0.44 (0.35–0.55) for deflazacort therapy. Excluding studies exhibiting any risk of bias (i.e., [49, 50], see Section 3.2 for details), the corresponding estimates were 0.46 (0.41–0.51) for glucocorticoid therapy (deflazacort/prednisone/prednisolone), 0.51 (0.44–0.59) for prednisone/prednisolone therapy, and 0.25 (0.18–0.35) for deflazacort therapy.

**Table 2:** Predictors of loss of ambulation in DMD

Author (year) [country]	Predictor(s)/indicator(s)	Definition of LoA	Method of analysis	Outcome results	Risk of bias
Barber et al. (2013) [US] [15]	Glucocorticoids (DFZ, PDN, or PRED)	Fulltime wheelchair use	Correlation analysis (method NR)	Correlation (duration of treatment and loss of ambulation): *r* = 0.3, *p* < 0.01.	Selection: ◊◊◊◊ Comparability: ◊◊ Outcome: ◊◊◊
Barnard et al. (2020) [US] [52]	Magnetic resonance biomarkers	Inability to perform the 10 m walk/run test	Discrete time hazard model	*Loss of ambulation at 12 months*	Selection: ◊◊◊ (case ascertainment NR) Comparability: ◊◊ Outcome: ◊◊ (duration of follow-up)
				•OR (1 SD increase in MRS FF VL): 10.8, *p*-value < 0.001 (SD: 0.20).
				•OR (1 SD increase in MRS FF SOL): 3.9, *p*-value < 0.001 (SD: 0.12).
				•OR (1 SD increase in MRI T_2_ VL): 4.4, *p*-value < 0.001 (SD: 11.8 ms).
				•OR (1 SD increase in MRI T_2_ BFLH): 3.8, *p*-value < 0.001 (SD: 13.6 ms).
Bello et al. (2015) [*] [12]	Glucocorticoids (DFZ, PDN, and/or PRED)	Continuous wheelchair use, confirmed by inability to walk 10 meter unaided	Kaplan-Meier (log-rank test)	*Median survival*	Selection: ◊◊◊◊ Comparability: ◊◊ Outcome: ◊◊◊
				•13 years (treated) vs. 10.0 years (untreated), *p* < 0.0001.
			Regression analysis (Cox proportional hazards model)	•HR (PDN/PRED vs. untreated): 0.498, 95% CI: 0.363–0.683, *p* < 0.001.
				•HR (DFZ vs. untreated): 0.294, 95% CI: 0.207–0.419, *p* < 0.001.
				•HR (daily regimen vs. untreated): 0.382, 95% CI: 0.285–0.515, *p* < 0.001.
				•HR (2 d/wk vs. untreated): 0.508, 95% CI: 0.301–0.856, *p* = 0.011.
				•HR (intermittent vs. untreated): 0.362, 95% CI: 0.190–0.689, *p* = 0.002.
Bello et al. (2015) [*] [16]	SPP1 rs28357094 genotype (TG, GG, and TT)	Continuous wheelchair use, confirmed by inability to walk 10 meter unaided	Kaplan-Meier (log-rank test)	*Median age at loss of ambulation* •11.8 years (TG/GG) vs. 13.0 years (TT), *p* = 0.048.	Selection: ◊◊◊◊ Comparability: ◊◊ Outcome: ◊◊◊
			Regression analysis (Cox proportional hazards model)	•HR (SPP1 rs28357094 [glucocorticoid-treated]): 1.61, *p* = 0.016.
	LTBP4 rs10880 genotype (CC, CT, and TT)		Kaplan-Meier (log-rank test)	*Median age at loss of ambulation (Caucasian cohort)*
				•12.6 years (CC/CT) vs. 15.0 years (TT), *p* = 0.024.
	Race/ethnicity (Caucasian, Hispanic-Caucasian, South Asian, Hispanic, Asian, African American, and other)			*Median age at loss of ambulation*
				•12.4 years (non-Hispanic) vs. 9.7 years (Hispanic), *p* = 0.003.
				•12.4 years (non-Hispanic) vs. 9.7 years (South Asian), *p* < 0.001.
	Glucocorticoid (agents NR)		Regression analysis (Cox proportional hazards model)	•HR (treatment vs. no treatment): 0.41, SE: 0.07, *p* < 0.001.
Bello et al. (2016) [*] [17]	*DMD* mutation	Continuous wheelchair use, confirmed by inability to walk 10 meter unaided	Regression analysis (Cox proportional hazards model)	•HR (Exon 44 skipping amenable deletion vs. other out-of-frame deletion): 0.34, 95% CI: 0.15–0.74, *p* = 0.007.	Selection: ◊◊◊◊ Comparability: ◊◊ Outcome: ◊◊◊
				•HR (deletion of exons 3–7 vs. other out-of-frame deletion): 0.24, 95% CI: 0.07–0.82, *p* = 0.02.
	Glucocorticoids (DFZ, PDN, or PRED)			•HR (PDN/PRED vs. no treatment): 0.34, 95% CI: 0.20–0.57, *p* = 0.0001.
				•HR (DFZ vs. no treatment): 0.22, 95% CI: 0.12–0.40, *p* = 0.0001.
Bello et al. (2016) [*] [18]	CD40 rs1883832 genotypes (CC, CT, and TT)^a^	NR	Regression analysis (Cox proportional hazards model)	•HR (CC vs. CT/TT^b^): 2.10, 95% CI: 1.45–3.04, *p* = 0.000035.	Selection: ◊◊◊◊ Comparability: ◊ (glucocorticoid exposure NR) Outcome: ◊◊◊
				•HR (CC vs. CT/TT^c^): 1.16, 95% CI: 1.02–1.32, *p* = 0.02.
	Glucocorticoid (agents NR)			•HR (treated vs. untreated^b^): 0.16, 95% CI: 0.09–0.29, *p* < 0.0001. •HR (treated vs. untreated^c^): 0.48, 95% CI: 0.40–0.58, *p* < 0.005.
Biggar et al. (2001) [CA] [19]	Glucocorticoid (DFZ)	Not able to walk with or without long leg braces on a level floor	Descriptive (Student’s *t*-test)	*Mean (SD) age at loss of ambulation*	Selection: ◊◊◊◊ Comparability: ◊◊ Outcome: ◊◊◊
				•12.3 years (2.7) (treated) vs. 9.8 years (1.8) years (untreated), *p* < 0.05.
Bonifati et al. (2006) [IT] [20]	Glucocorticoids (DFZ and/or PDN)	NR	Regression analysis (Cox proportional hazards model)	•HR (treated early vs. NR): NR, t: –4.63; *p* = 0.00004.	Selection: ◊◊◊◊ Comparability: ◊◊ Outcome: ◊◊◊
				•HR (long treatment duration vs. NR): NR, t: –4.63; *p* = 0.00004.
Chen et al. (2020) [CN] [21]	Glucocorticoids (DFZ or PDN)	Continuous wheelchair use, confirmed by inability to walk 10 meter unaided	Kaplan-Meier (log-rank test)	*Median age at loss of ambulation*	Selection: ◊◊◊◊ Comparability: ◊◊ Outcome: ◊◊◊
				•11.67 years (treated) vs. 9.92 years (untreated), *p* < 0.001.
	*DMD* mutation (truncated and non-truncated)			*Median age at loss of ambulation*
				•10.42 years (truncated mutations) vs. 13.17 years (non-truncated mutations), *p* < 0.001.
	SPP1 rs11730582 genotype (CC, CT, and TT)			*Median age at loss of ambulation (truncated DMD)*
				•12.00 years (CC/CT) vs. 10.67 years (TT), *p* = 0.006.
			Regression analysis (Cox proportional hazards model)	•HR (CC/CT vs. TT): 0.63, 95% CI: 0.45–0.89, *p* = 0.008.
Ciafaloni et al. (2016) [US] [22]	Age at onset of signs or symptoms	Full-time wheelchair use or ceased ambulation	Regression analysis (Cox proportional hazards model)	HR (age at onset of signs or symptoms): 0.90, 95% CI: 0.87–0.94, *p* < 0.0001.	Selection: ◊◊◊◊ Comparability: ◊◊ Outcome: ◊◊◊
Fischmann et al. (2012) [CH] [53]	Magnetic resonance biomarkers	<25% for the D1 subscale of the MFM and patient report	Correlation analysis (Spearman’s correlation coefficient [*ρ*])	•MRI FF left quadriceps: *ρ*= 0.93, *p* < 0.001.	Selection: ◊◊◊◊ Comparability: ◊ (glucocorticoid exposure NR) Outcome: ◊◊ (duration of follow-up)
				•MRI FF right quadriceps: *ρ*= 0.91, *p* < 0.001.
				•MRI FF left hamstrings: *ρ*= 0.86, *p* < 0.001.
				•MRI FF right hamstrings: *ρ*= 0.85, *p* < 0.001
			NR	S*ensitivity and specificity for predicting LoA (at 50% cut-off for MRI FF)*
				•Left leg: 100% and 91%, *p* < 0.0001.
				•Right leg: 100% and 100%, *p* < 0.0001.
Flanigan et al. (2013) [US] [23]	LTBP4 genotype	NR	Regression analysis (Cox proportional hazards model)	•HR (rs10880 genotype): 0.52, 95% CI: 0.34–0.78, *p* = 0.001.	Selection: ◊◊◊◊ Comparability: ◊◊ Outcome: ◊◊◊
	Glucocorticoids (agents NA)			•HR (treated vs. untreated): 0.65, 95% CI: 0.50–0.84, *p* = 0.001.
Godi et al. (2016) [IT] [54]	Magnetic resonance biomarkers	NR	ROC curves	•Accuracy for discriminating between ambulatory and non-ambulatory patients: SIR quadriceps (92%), SIR flexors (96%), thigh MVI (92%), calf MVI (85%), biceps MVI (96%), and soleus MVI (100%) (all *p* < 0.05).	Selection: ◊◊◊◊ Comparability: ◊◊ Outcome: ◊◊ (duration of follow-up)
Haber et al. (2021) [US] [24]	*DMD* mutation	NR	Kaplan-Meier (log-rank test)	•Survival function (exon 8 skippable vs. all other deletions): χ^2^: 9.68, *p* < 0.01.	Selection: ◊◊◊◊ Comparability: ◊◊ Outcome: ◊◊◊
				•Survival function (exon 44 skippable vs. all other deletions): χ^2^: 5.05, *p* < 0.05.
			Regression analysis (Cox proportional hazards model)	•HR (exon 8 skippable vs. other exon skippable): 0.22, 95% CI: 0.08–0.63, *p* < 0.05.
				•HR (exon 44 skippable vs. other exon skippable): 0.30, 95% CI: 0.12–0.78, *p* < 0.05.
				•HR (exon 45 skippable vs. exon 8 skippable): 5.80, 95% CI: 1.07–31.41, *p* < 0.05.
				•HR (exon 51 skippable vs. exon 8 skippable): 5.28, 95% CI: 1.01–27.66, *p* < 0.05.
	Glucocorticoids (DFZ and/or PDN)			•HR (treatment vs no treatment): 0.72, 95% CI: 0.53–0.97, *p* < 0.05.
	Age at onset of signs or symptoms			•HR (age at onset of signs or symptoms): 0.89, 95% CI: 0.84–0.95, *p* < 0.05.
Houde et al. (2008) [CA] [25]	Glucocorticoids (DFZ)	NR	Descriptive (Student’s *t*-test)	*Mean (SD) age at loss of ambulation*	Selection: ◊◊◊◊ Comparability: ◊◊ Outcome: ◊◊◊
				•11.5 years (1.9) (treated) vs. 9.6 years (1.4) (untreated), *p* < 0.05.
Hufton et al. (2017) [UK] [26]	Race/ethnicity (white British and South Asian)	NR	Kaplan-Meier (log-rank test)	*Mean (95% CI) age at loss of ambulation*	Selection: ◊◊◊◊ Comparability: ◊◊ Outcome: ◊◊◊
				•138.7 months (121.4–156 months) (white British heritage) vs. 115.2 months (103.6–126.7 months) (South Asian heritage), *p* < 0.05.
	Deprivation (top 20%) [Townsend deprivation index decile 1 and 2]) and bottom 20% [Townsend deprivation index decile 9 and 10]			*Mean age at loss of ambulation*
				•130.0 months (top 20%) vs. 102.5 months (lowest 20%), *p* < 0.05.
Humbertclaude et al. (2012) [FR] [27]	Age of scoliosis diagnosis	NR	Correlation analysis (Spearman’s correlation coefficient [*ρ*])	*ρ*= 0.45, *p* < 0.0001	Selection: ◊◊◊◊ Comparability: ◊◊ Outcome: ◊◊◊
	Age at loss of running ability			*ρ*= 0.85, *p* < 0.0001
	Age at loss of climbing stairs			*ρ*= 0.84, *p* < 0.0001
	Age at loss of rising from the floor			*ρ*= 0.91, *p* < 0.0001
	Age at loss of sitting up by himself			*ρ*= 0.85, *p* < 0.0001
	Age at loss of seated position without support			*ρ*= 0.60, *p* < 0.0001
	Age at loss of raising the hand up to the head			*ρ*= 0.60, *p* < 0.0001
Kim et al. (2015) [US] [28]	Glucocorticoids (DFZ and/or PDN)	NR	Descriptive (Student’s *t*-test)	*Mean (SE) age at loss of ambulation*	Selection: ◊◊◊◊ Comparability: ◊◊ Outcome: ◊◊◊
				•12.3 years (0.2) (long treatment duration) vs. 10.3 years (0.1) (untreated), *p* < 0.05.
	Regression analysis (Cox proportional hazards model)			•HR (short treatment vs no treatment): 1.77, 95% CI: 1.34–2.32, *p* < 0.001.
				•HR (long treatment vs. no treatment) (≤11 years): 0.18, 95% CI: 0.10–0.29, *p* < 0.001.
King et al. (2007) [US] [29]	Glucocorticoids (DFZ and/or PDN)	Loss of independent ambulation (defined as functional walking without orthoses or any assistive device)	Descriptive (Student’s *t*-test)	*Mean (SD) age at loss of ambulation*	Selection: ◊◊◊◊ Comparability: ◊◊ Outcome: ◊◊◊
				•12.52 years (3.02) (treated) vs. 9.21 years (1.48) (untreated), *p* < 0.0001.
Koeks et al. (2017) (*) [30]	Glucocorticoids (DFZ, PDN, or PRED)	Wheelchair dependence	Turnbull analysis (test NR)	*Median age at loss of ambulation*	Selection: ◊◊◊◊ Comparability: ◊◊ Outcome: ◊◊◊
				•13 years (treated) vs. 10 years (untreated), *p* < 0.001. *Median survival*
				•14 years (treated) vs. 8 years (previously treated) vs. 10 years (never treated), *p* < 0.05
Kosac et al. (2022) [RS] [31]	Glucocorticoids (DFZ or PRED)	Inability to walk 10 m independently	Kaplan-Meier (log-rank test)	*Mean (95% CI) age at loss of ambulation*	Selection: ◊◊◊◊ Comparability: ◊◊ Outcome: ◊◊◊
				•11.14 years (10.52–11.76) (treated) vs. 9.95 years (8.9–11) (untreated), *p* = 0.021.
			Regression analysis (Cox proportional hazards model)	HR (treated vs. untreated): 0.44, 95% CI: 0.23–0.83, *p* = 0.01.
	DMD mutation (“proximal”, [mutation upstream intron 44], vs. “distal” [mutation intron 44 and downstream])		Kaplan-Meier (log-rank test)	*Mean (95% CI) age at loss of ambulation (glucocorticoid-naive)*
				•10.88 years (8.51–13.25) (proximal) vs. 9.27 years (8.40–10.14) (distal), *p* = 0.013.
			Regression analysis (Cox proportional hazards model)	HR (distal vs. proximal): 1.92, 95% CI: 1.07–3.47, *p* = 0.03.
Mazzone et al. (2013) [IT] [32]	NSAA (score≤22 vs. >22)^d^	NR	Regression analysis (logistic model)	OR: 37.5, 95% CI: 4.7–300.4, *p* = 0.001.	Selection: ◊◊◊◊ Comparability: ◊◊ Outcome: ◊◊◊
	6MWT (≤330 vs. >330 m)^d^			OR: 23.6, 95% CI: 4.9–113.8, *p* < 0.001.
	Gowers test (≤7.2 vs. >7.2 s)^d^			OR: 6.2, 95% CI: 1.6–23.6, *p* = 0.007.
	10 m timed test (≤7 vs. >7 s)^d^			OR: 7.9, 95% CI: 2.2–28.3, *p* = 0.002.
McDonald et al. (2018) [*] [33]	Glucocorticoids (DFZ, PDN, or PRED)	NR	Kaplan-Meier (log-rank test)	*Median (95% CI) age at loss of ambulation*	Selection: ◊◊◊◊ Comparability: ◊◊ Outcome: ◊◊◊
				•13.40 years (12.50–14.00) (≥1 year of treatment) vs. 10.00 years (9.30–10.80) (<1 year of treatment), *p* < 0.0001.
				•11.30 years (11.00–13.00) (PDN/PRED) vs. 14.00 years (13.30–15.00) (DFZ), *p* = 0.0102.
McDonald et al. (2022) [*] [34]	Ataluren	Study-specific (see article for details)	Kaplan-Meier (log-rank test)	*Median age at loss of ambulation*	Selection: ◊◊◊◊ Comparability: ◊ (indirect comparison) Outcome: ◊◊◊
				•15.5 years (ataluren) vs. 13.3 years (CINRG DNHS), *p* = 0.0006.
Mendell et al. (2016) [US] [35]	Eteplirsen	NR	Descriptive (test NR)	*Proportion non-ambulatory after 3 years*	Selection: ◊◊◊◊ Comparability: ◊ (indirect comparison) Outcome: ◊◊◊
				•16.7% (treated) vs. 46.2% (untreated), *p* < 0.05
Mendell et al. (2021) [US] [36]	Eteplirsen	0 meters on the 6MWT	Kaplan-Meier (log-rank test)	*Proportion ambulatory after 4 years*	Selection: ◊◊◊◊ Comparability: ◊ (indirect comparison) Outcome: ◊◊◊
				•73% (treated) vs. 17% (untreated), *p* = 0.020.
Mercuri et al. (2020) [*] [37]	Ataluren	NR	Kaplan-Meier (log-rank test)	*Median (95% CI) age at loss of ambulation*	Selection: ◊◊◊◊ Comparability: ◊ (indirect comparison) Outcome: ◊◊◊
			Regression analysis (Cox proportional hazards model)	•HR (treated vs. untreated): 0.283, 95% CI: 0.190–0.422, *p* < 0.05.
				•14.5 years (13.9-NA) vs. 11.0 years (10.5–12.0), *p* < 0.0001.
Mitelman et al. (2022) [*] [38]	Eteplirsen	Study-specific (see article for details)	Kaplan-Meier (log-rank test)	*Medan time from baseline to loss of ambulation*	Selection: ◊◊◊◊ Comparability: ◊ (indirect comparison) Outcome: ◊◊◊
				•5.09 years (eteplirsen) vs. 3.00 (untreated), *p* < 0.01.
			Regression analysis (Cox proportional hazards model)	HR (treated vs. untreated): 0.119, 95% CI: 0.016–0.863, *p* < 0.05.
Naarding et al. (2020) [NL] [55]	Magnetic resonance biomarkers	Unable to walk 5 m without assistance or orthoses	Regression analysis (Cox proportional hazards model)	HR (MRI FF VL %): 1.15, 95% CI: 1.05–1.26, *p* = 0.003.	Selection: ◊◊◊◊ Comparability: ◊◊ Outcome: ◊◊◊
			Correlation analysis (Spearman’s correlation coefficient [*ρ*])	*ρ* (MRI FF VL) = –0.72, *p* < 0.001.
Pane et al. (2014) [IT] [39]	6MWT	NR	Descriptive (χ^2^ test)^b^	*Proportion non-ambulatory after 36 months*	Selection: ◊◊◊◊ Comparability: ◊◊ Outcome: ◊◊◊
				•52.94% (6MWT < 350 metres) vs. 9.68% (6MWT≥350 metres), *p* < 0.001.
Rooney et al. (2020) [US] [56]	Magnetic resonance biomarkers	Inability to perform the 10 m walk/run test	Regression analysis (Cox proportional hazards model)	*1-year decrement in the average age at half-maximal muscle involvement* •HR (MRS FF VL): 2.43, 95% CI: 1.77–3.35, *p* < 0.000001. •HR (MRS FF SOL): 2.25, 95% CI: 1.65–3.05, *p* < 0.0000001. •HR (MRS FF VL SOL [composite measure]): 2.71, 95% CI: 1.92–3.81, *p* < 0.0000001.	Selection: ◊◊◊◊ Comparability: ◊◊ Outcome: ◊◊◊
Schara et al. (2001) [DE] [40]	Glucocorticoids (DFZ)	NR	Descriptive (Fisher’s exact test)	*Proportion ambulatory at end of follow-up* •100% (treated) vs. 0% (untreated), *p* < 0.0001.	Selection: ◊◊◊ (case ascertainment NR) Comparability: ◊◊ Outcome: ◊◊◊
Servais et al. (2015) [FR] [41]	DMD mutations (deletions treatable by exon 53 skipping [DMD-53]; mutations not treatable by exon 53 skipping [DMD all-non-53]; and deletions not treatable by exon 53 skipping [DMD del-non-53])	NR	Descriptive (Mann-Whitney *U* test)	*Mean (SD) age at loss of ambulation* •8.7 years (1.6) (DMD-53) vs. 10.4 years (2.4) (DMD-all-non-53), *p* = 0.031. •8.7 years (1.6) (DMD-53) vs. 10.7 years (2.1) (DMD-del-non-53), *p* = 0.011.	Selection: ◊◊◊◊ Comparability: ◊◊ Outcome: ◊◊◊
Sherlock et al. (2022) [*] [57]	Magnetic resonance biomarkers	Gait inability	Regression analysis (Cox proportional hazards model)	•HR (total thigh MVI below median baseline value): 6.3, 95% CI: 2.0–20.4, *p* = 0.002. •HR (mean FF muscle bundle): 5.8, 95% CI: 1.5–22.2, *p* = 0.010. •HR (mean FF lean muscle): 3.9, 95% CI: 1.2–5.2, *p* = 0.029.	Selection: ◊◊◊◊ Comparability: ◊◊ Outcome: ◊◊ (duration of follow-up)
Silversides et al. (2003) [CA] [42]	Glucocorticoids (DFZ)	NR	Descriptive (Fisher’s exact test)	*Proportion non-ambulatory at end of follow-up* •48% (treated) vs. 100% (untreated), *p* = 0.002.	Selection: ◊◊◊◊ Comparability: ◊◊ Outcome: ◊◊◊
Spitali et al. (2020) [*] [43]	*TCTEX1D1* rs1060575 and rs3816989 genotypes (AA, AT, and TT)	NR	Regression analysis (Cox proportional hazards model)	Survival functions (TT vs. AA/AT), *p* = 0.032	Selection: ◊◊◊◊ Comparability: ◊ (glucocorticoid exposure NR) Outcome: ◊◊◊
Takeuchi et al. (2013) [JP] [44]	Glucocorticoids (PRED)	Loss of independent walking (defined as unsupported walking indoors)	Kaplan-Meier (log-rank test)	*Median (IQR) age at loss of ambulation* •132 months (126–138) (treated) vs. 121 months (120–126) (untreated), *p* = 0.0002.	Selection: ◊◊◊◊ Comparability: ◊◊ Outcome: ◊◊◊
			Regression analysis (Cox proportional hazards model)	HR (treated vs untreated): 0.64, 95% CI: 0.50–0.82, *p* = 0.0005.
van den Bergen et al. (2014) [NL] [46]	Glucocorticoids (agents NR)	NR	Kaplan-Meier (log-rank test)	*Median age at loss of ambulation* •11.6 years (treated) vs. 9.8 years (untreated), *p* < 0.001.	Selection: ◊◊◊◊ Comparability: ◊◊ Outcome: ◊◊◊
van den Bergen et al. (2014) [NL] [45]	*DMD* mutation	NR	Kaplan-Meier (log-rank test)	*Mean age at loss of ambulation* •10.8 years (exon 44 skippable) vs. 9.8 years (other skippable), *p* = 0.020.	Selection: ◊◊◊◊ Comparability: ◊◊ Outcome: ◊◊◊
van den Bergen et al. (2015) [†] [47]	Glucocorticoids (agents NR)	NR	Kaplan-Meier (log-rank test)	*Mean age at loss of ambulation* •11.9 (treated) vs. 9.6 (untreated), *p* < 0.001.	Selection: ◊◊◊◊ Comparability: ◊◊ Outcome: ◊◊◊
			Regression analysis (Cox proportional hazards model)	*SPP1* •HR (treated vs. untreated): 0.38, *p* < 0.001. *LTBP4* •HR (treated vs. untreated): 0.31, *p* < 0.001.
	Haplotypes of the LTBP4 gene (IAAM, VTTT, other)			HR (IAAM vs. VTTT): 0.8, *p* = 0.046.
	LTBP4 diplotypes (IAAM/IAAM, other)			HR (IAAM/IAAM vs. other)): 1.30, *p* = 0.01.
	Country of residence (NL, IT, and UK)			•HR (IT vs. UK): 1.62, *p* = 0.03. •HR: (NL vs. UK): 1.83, *p* = 0.005.
Vry et al. (2016) [‡] [48]	Glucocorticoids (agents NR)	NR	Kaplan-Meier (log-rank test)	*Median (95% CI) age at loss of ambulation* •10.08 (9.58–10.50) (treated) vs. 11.42 (10.45–11.50) (untreated), *p* < 0.05.	Selection: ◊◊◊◊ Comparability: ◊◊ Outcome: ◊◊◊
Wang et al. (2014) [US] [50]	Glucocorticoids (DFZ and/or PDN)	Self-reported	Kaplan-Meier (log-rank test)	*Median age at loss of ambulation* •13 years (treated) vs. 10 years (untreated/previously treated), *p* < 0.0001. •14 years (DFZ) vs. 13 years (PDN), *p* = 0.0013.	Selection: ◊◊◊ (case ascertainment NR) Comparability: ◊◊ Outcome: ◊◊ (patient-reported)
			Regression analysis (Cox proportional hazards model)	HR (treated vs. untreated/previously treated): 0.35, 95% CI: 0.28–0.43, *p* < 0.0001.
				HR (DFZ vs. no treatment): 0.68, 95% CI: 0.51–0.92, *p* < 0.05.
	Vitamin D		Kaplan-Meier (log-rank test)	*Proportion ambulatory at 12 years of age* •72% (glucocorticoids and vitamin D) vs. 54% (glucocorticoids), *p* = 0.004.
	Coenzyme Q10			*Proportion ambulatory at 12 years of age* •74% (glucocorticoids and coenzyme Q10) vs. 54% (glucocorticoids), *p* = 0.007.
			Regression analysis (Cox proportional hazards model)	HR (coenzyme Q10): 0.68, 95% CI: 0.47–0.98, *p* < 0.05.
Wang et al. (2018) [US] [49]	*DMD* mutation	NR	Kaplan-Meier (log-rank test)	*Median age at loss of ambulation* •20 years (exon 44 skippable) vs. 13 years (other mutations), *p* = 0.035. *Proportion ambulatory at 20 years of age* •95% (exon 8 skippable) vs. 8% (other mutations), *p* < 0.00001. *Median age at loss of ambulation* •12 years (exon 51 skippable) vs. 13 years (other mutations), *p* = 0.035. *Proportion ambulatory at 15 years of age* •63% (single exon 45 deletions) vs. 33% (other mutations), *p* = 0.029. *Proportion ambulatory at 15 years of age* •30% (exon 49–50 deletions) vs. 35% (other mutations), *p* = 0.00791. *Proportion ambulatory at 20 years of age* •95% (exon 3–7 deletions) vs. 8% (other mutations), *p* = 0.0003.	Selection: ◊◊◊ (case ascertainment NR) Comparability: ◊ (glucocorticoid exposure NR) Outcome: ◊◊ (patient-reported)
			Regression analysis (Cox proportional hazards model)	•HR (exon 8 skippable vs. other): 0.21, 95% CI: 0.08–0.53, *p* < 0.01. •HR (exon 44 skippable vs. other): 0.54, 95% CI: 0.33–0.87, *p* = 0.01.
	Glucocorticoids (DFZ and/or PDN)			•HR (DFZ vs. no treatment): 0.31, 95% CI: 0.22–0.43, *p* < 0.01. •HR (PDN vs. no treatment): 0.62, 95% CI: 0.44–0.88, *p* < 0.01.
Yılmaz et al. (2004) [TR] [51]	Glucocorticoids (PRED)	NR	Descriptive (Student’s *t*-test)	*Mean (SD) age at loss of ambulation* •10.0 years (1.5) (treated) vs. 8.6 years (2.6) (untreated), *p* < 0.05.	Selection: ◊◊◊ (case ascertainment NR) Comparability: ◊◊ Outcome: ◊◊◊
Zhang et al. (2021) [CN] [13]	Glucocorticoids (DFZ, PDN, and/or PRED)	NR	Regression analysis (Cox proportional hazards model)	•HR (DFZ vs. no treatment): 0.06, 95% CI: 0.02–0.19, *p* < 0.001. •HR (PDN/PRED vs. no treatment): 0.40, 95% CI: 0.31–0.52, *p* < 0.001.	Selection: ◊◊◊◊ Comparability: ◊◊ Outcome: ◊◊◊
	*DMD* mutation			•HR (nonsense mutations vs. other deletions): 0.66, 95% CI: 0.44–0.99), *p* = 0.045. •HR (Exon 44 amenable skipping vs. other deletions): 0.56, 95% CI: 0.33–0.94, *p* = 0.029.

Note: Biceps femoris long head (BFLH). Canada (CA). China (CN). Confidence interval (CI). Deflazacort (DFZ). Duchenne muscular dystrophy (DMD). Fat fraction (FF). France (FR). Germany (DE). Hazard ratio (HR). Italy (IT). Japan (JP). Magnetic resonance imaging (MRI). Magnetic resonance spectroscopy (MRS). Muscle volume index (MVI). Not applicable (NA). Not reported (NR). Prednisolone (PRED). Prednisone (PDN). Serbia (RS). Signal intensity ratio (SIR). Soleus (SOL). Standard deviation (SD). Switzerland (CH). The Netherlands (NL). Turkey (TR). United Kingdom (UK). United States of America (US). Vastus lateralis (VL). Risk of bias was assessed using the Newcastle–Ottawa Scale. Maximum score: ◊◊◊◊ for selection, ◊◊ for comparability, and ◊◊◊ for outcome (see the Methods section for details). *Multi-national (see article for details). †The Netherlands, Italy, France, and the United Kingdom. ‡Bulgaria, the Czech Republic, Denmark, Germany, Hungary, Poland, and the United Kingdom. ^a^Due to low statistical power in the initial GWAS, the authors hypothesized that some nominally significant *p* values may indicate true associations, indeed they focus their analysis on SNPs lying within, or less than 10 Kb upstream or downstream of genes implicated in DMD-related pathways (i.e., the nuclear factor kB [NF-kB] and transforming growth factor b [TGFb] signaling pathways). Full gene table available as supplemental material to the original article online. ^b^The CINRG Exome Chip cohort (see article for details). ^c^Overall validation cohort (see article for details). ^d^At baseline (maximum follow-up: 24 months).

Bello et al. [12] investigated the effectiveness of different glucocorticoid regimens (agents not reported) in a prospective cohort study of 340 children and adults with DMD (mean age: 14 years; multi-national). The HR for loss of ambulation (vs. no glucocorticoid treatment) was estimated at 0.38 for the daily regimen (*p* < 0.001), 0.51 for the weekend-regimen (*p* = 0.011), and 0.36 for the intermittent regimen (*p* = 0.002).

Moreover, in a retrospective cohort study of 336 patents with DMD (mean age not reported), van den Bergen et al. [47] reported estimates of the effect of glucocorticoids (agents not reported) in patient with *SPP1* and *LTBP4* polymorphisms, respectively. The HR for loss of ambulation (vs. no glucocorticoid treatment) in each group was estimated at 0.38 and 0.31, respectively (both *p* < 0.001).

Barber et al. [15] studied the association between duration of treatment with deflazacort, prednisone, or prednisolone and age at fulltime wheelchair use 

among 462 US patients with DMD (mean age not reported). The estimated correlation coefficient (analysis method not reported) was *r* = 0.3, *p* < 0.01.

Finally, in a retrospective cohort study of 477 US patents with DMD (mean age: 7 years), Kim et al. [28] found that, compared with no glucocorticoid treatment, patients treated for a short duration had a higher risk of becoming non-ambulatory (HR: 1.77, 95% CI: 1.34–2.32, *p* < 0.001) and patients treated for a long duration had a lower risk (HR: 0.18, 95% CI: 0.10–0.29, *p* < 0.001).

#### Age at onset of signs or symptoms

We identified two retrospective cohort studies reporting evidence of predictive effects of age at onset of signs or symptoms on loss of ambulation in DMD (Table 2). Specifically, in their work based on the MD STAR*net* database (US, multi-centre), encompassing 825 patients with DMD (mean age not reported), Ciafaloni et al. [22] estimated the HR for loss of ambulation for age at onset of signs or symptoms (i.e., trouble rising/Gowers’ sign, trouble walking/running/jumping, frequent falling/clumsiness, inability to keep up with peers, abnormal gait, loss of motor skills, gross motor delay, or muscle weakness) at 0.90 (*p* < 0.0001). Almost identical results were reported by Haber et al. [24] in their study of 358 adult patients with DMD based on data from the same database but covering a slightly longer time period (HR: 0.90, *p* < 0.05).

#### Developmental milestones and functional ability

We identified three observational studies reporting evidence of predictive effects of developmental milestones and functional ability on loss of ambulation in DMD (Table 2). Specifically, Humbertclaude et al. [27] studied motor and respiratory heterogeneity in a sample of 278 French patients with DMD (mean age: 11 years) and found a range of motor milestones to be significantly associated with age at loss of ambulation, including age at loss of running ability (Spearman’s Rho [*ρ*] = 0.85), age at loss of climbing stairs (*ρ*= 0.84), age at loss of rising from the floor (*ρ*= 0.91), age at loss of sitting up by himself (*ρ*= 0.85), age at loss of seated position without support (*ρ*= 0.60), and age at loss of raising the hand up to the head (*ρ*= 0.60), as well as age at scoliosis diagnosis (*ρ*= 0.45) (all *p* < 0.0001). Moreover, in their prospective cohort study encompassing 96 Italian children with DMD (mean age: 8 years), Pane et al. [39] estimated the proportion of patients who were non-ambulatory after 36 months at 53% for those with a baseline six-minute walk test (6MWT) result < 350 metres, and at 10% for patients who were able to walk≥350 metres (*p* < 0.001). Finally, Mazzone et al. [32] studied the impact of baseline function on the risk of becoming non-ambulatory within 24 months among 113 Italian children with DMD (mean age: 8 year). The authors found that patents with a baseline NSAA score equal to or below 22 (vs. >22) had a significantly higher risk of becoming non-ambulatory across follow-up (OR: 37.5, *p* = 0.001). Similar findings were noted for the 6MWT (≤330 vs. >330 meters) (OR: 23.6, *p* < 0.001), Gowers test (≤7.2 vs. >7.2 s) (OR: 6.2, *p* = 0.007), and the 10 meter timed test (≤7 vs. >7 s) (OR: 7.9, *p* = 0.002).

#### DMD mutations and genetic modifiers

**Fig. 4:**
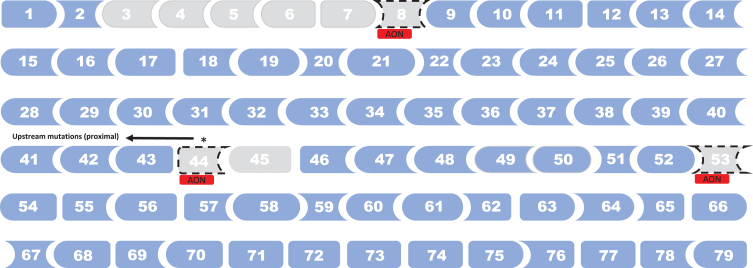
*DMD* mutations spots associated with later loss of ambulation in DMD. Note: Deletion of exons 3–7 vs. other out-of-frame deletion [16, 48]. Exon 8 skipping amenable deletion vs. other exon skippable [23, 48]. Proximal (mutation upstream intron 44) vs. distal (mutation intron 44 and downstream) [30]. Exon 44 skipping amenable deletion vs. other out-of-frame deletion or other exon skippable [16, 23, 44, 48, 51]. Single exon 45 deletions vs. other mutations [48]. Exon 53 skipping amenable deletion vs. other deletion [40]. Nonsense mutations vs. other deletions were also associated with later loss of ambulation [51] (not shown in figure).

**Fig. 5:**
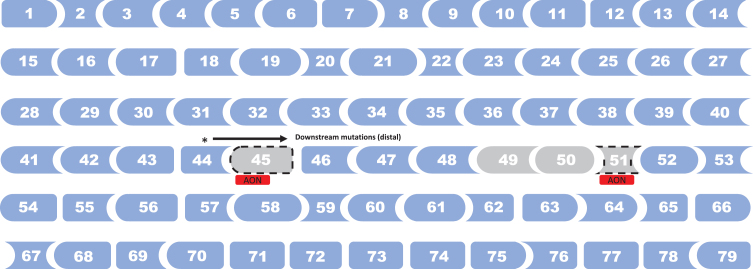
*DMD* mutations spots associated with earlier loss of ambulation in DMD. Note: Distal (mutation intron 44 and downstream) vs. proximal (mutation upstream intron 44) [30]. Exon 45 skipping amenable deletion vs. exon 8 skipping amenable deletion [23]. Deletion of exons 49–50 vs. other mutations [48]. Exon 51 skipping amenable deletion vs. other deletion [48], and exon 51 skipping amenable deletion vs. exon 8 skipping amenable deletion [23].

We identified 13 observational studies reporting evidence of effects of *DMD* mutations or genetic modifiers on loss of ambulation in DMD [13, 16–18, 21, 23, 24, 31, 41, 43, 45, 47, 49] (Table 2, Table 3). *DMD* mutations spots associated with later loss of ambulation in DMD are illustrated in Fig. 4, and with earlier loss of ambulation in Fig. 5. In the prospective cohort study by Bello et al. [17], involving 212 patients with DMD (multi-national cohort; mean age: not reported), deletions amenable to skipping of exon 44 and exons 3–7 were found to be associated with prolong ambulation compared with other out-of-frame deletion (HR: 0.34, *p* = 0.007; and HR: 0.24, *p* = 0.02, respectively). DMD patients with nonsense mutation showed a typical median age at loss of ambulation (11.1 years), with a few outliers (ambulatory around or after 16 years of age) carrying stop codons within in-frame exons, more often situated in the rod domain.

**Table 3:** Summary of genetic modifiers associated with loss of ambulation in patients with DMD

	Gene	SNP	Allele or haplotype	Minor allele or haplotype	*P* value
Bello et al. (2016) [18]	*CD40*	rs1883832	CT/CC	TT	0.000035
Bello et al. (2015) [16]	*LTBP4*	rs10880	CC/CT	TT	0.024
Ciafaloni et al. (2016) [22]	*LTBP4*	rs10880	NA	NA	<0.001
van den Bergen et al. (2015) [47]	*LTBP4*	–	IAAM	VTTT	0.046
		–	IAAM/IAAM	Others	0.01
Bello et al. (2015) [16]	*SPP1*	rs2835709	TG/GG	TT	0.048
Chen et al. (2020) [21]	*SPP1*	rs11730582	CC/CT	TT	0.006
Spitali et al. (2020) [43]	*TCTEX1D1*	rs1060575	AA/AT	TT	0.032

Note: Duchenne muscular dystrophy (DMD).

In a retrospective cohort study based on the MD STAR*net* database (US, multi-centre), involving 358 patients with DMD (mean age not reported), Haber et al. [24] studied the impact of mutation type on loss of ambulation. The HR for exon 8 skippable mutations (vs. other exon skippable mutations) was estimated at 0.22, for exon 44 skippable mutations (vs. other exon skippable mutations) at 0.30, for exon 45 skippable mutations (vs. exon 8 skippable mutations) at 5.80, for exon 51 skippable mutations (vs. exon 8 skippable mutations) at 5.28 (all *p* < 0.05).

In a case series of 35 French patients with DMD (mean age: 14 years), Servais et al. [41] investigated the clinical and functional status of patients theoretically treatable by exon 53 skipping. The mean age at loss of ambulation was estimated at 8.7 years for participants with deletions treatable by exon 53 skipping, compared with 10.4 for those with mutations not treatable by exon 53 skipping (*p* = 0.031) and 10.7 years for those with deletions not treatable by exon 53 skipping (*p* = 0.011).

In a study of 967 Chinese patients with DMD (mean age not reported), of which 55% were treated with glucocorticoids, Zhang et al. [13] found that nonsense mutations versus other deletions were associated with prolonged ambulation (HR: 0.66, *p* = 0.045), as well as exon 44 amenable skipping versus other deletions (HR: 0.56, *p* = 0.029).

Wang et al. [49] investigated correlation between mutation subgroups and patient-reported age at loss of ambulation in a sample of 765 US patients with DMD (mean age not reported). Participants amendable to exon 44 and exon 8 skipping showed prolonged ambulation compared to other exon skip groups and nonsense mutations (HR: 0.21, *p* < 0.01, and HR: 0.54, *p* = 0.01, respectively). The median age at loss of ambulation was 12 years for exon 51 skippable and 13 years for other mutations (*p* = 0.035). The proportion of patients still ambulatory at 15 years of age was estimated at 63% for patients with single exon 45 deletions and 33% for those with other mutations (*p* = 0.029) and at 30% for patients with exon 49–50 deletions and 35% for those with other mutations (*p* = 0.00791). The proportion of patients still ambulatory at 20 years of age was estimated at 95% for patients with exon 3–7 deletions and 8% for those with other mutations (*p* = 0.0003).

van den Bergen et al. [45] studied the impact of deletions treatable by exon 44 skipping in a sample comprising 114 Dutch patients with DMD (mean age not reported). The mean age at loss of ambulation was estimated at 10.8 years for participants with deletions treatable by exon 44 skipping and 9.8 for those with mutations not treatable by exon 53 skipping (*p* = 0.020).

Kosac et al. [31] investigated the effect of the *SPP1*, *CD40*, and *LTBP4* genes and *DMD* mutation location on loss of ambulation among 95 Serbian patients with DMD (mean age: 16 years). The mean age at loss of ambulation among glucocorticoid-naïve participants was estimated at 10.88 years for those with proximal mutations and 9.27 years for distal mutations (*p* = 0.013). The corresponding HR (distal vs. proximal) was estimated at 1.92 (*p* = 0.03).

Bello et al. [16] examined the effects of *LTBP4* and *SPP1* polymorphisms on age at loss of ambulation in a multi-ethnic cohort comprising of 340 patients with DMD part of the Cooperative International Neuromuscular Research Group Duchenne Natural History Study (CINRG DNHS). For the *SPP1* rs28357094 genotype, median age at loss of ambulation was estimated at 11.8 years for TG/GG and 13.0 years for TT (*p* = 0.048), and at 12.6 years (CC/CT) vs. 15.0 years (TT), *p* = 0.024. Corresponding estimates for the *LTBP4* rs10880 genotype were 12.6 years for CC/CT and 15.0 years for TT (*p* = 0.024). Among glucocorticoid-treated participants, *SPP1* rs28357094 was found to be associated with a higher risk of loss of ambulation (HR: 1.61, *p* = 0.016).

Chen et al. [21] studied *LTBP4* haplotypes and the *SPP1* promoter SNPs rs28357094, rs11730582, and rs17524488 in 326 Chinese children with DMD (mean age: 8 years). For the *SPP1* rs11730582 genotype, median age at loss of ambulation was estimated at 12.00 years for CC/CT and 10.67 years for TT (*p* = 0.006), corresponding to a HR of 0.63 (*p* = 0.008). Moreover, median age at loss of ambulation was shorter for truncated compared with non-truncated mutation (10.42 years vs. 13.17 years, *p* < 0.001).

Flanigan et al. [23] studied the *LTBP4* genotype and DMD severity in a cohort comprising of 239 US patients with DMD (mean age not reported). The HR for the rs10880 genotype (vs. other genotypes) was estimated at 0.52 (*p* = 0.001).

In their retrospective cohort study of 336 European patients (mean age not reported), van den Bergen et al. [47] found that the IAAM haplotype of the *LTBP4* gene was associated with prolonged ambulation compared with the VTTT haplotype (HR: 0.8, *p* = 0.046). In contrast, the IAAM/IAAM *LTBP4* diplotypes were associated with a higher risk compared with other diplotypes (HR: 1.3, *p* = 0.01).

In their retrospective cohort study involving 437 multi-national patients with DMD, Spitali et al. [43] found that age at loss of ambulation was significantly different between the *TCTEX1D1* rs3816989 and rs1060575 TT genotype and the AA/AT genotypes (*p* = 0.032).

Finally, Bello et al. [18] studied the impact of *CD40* rs1883832 genotype (CC, CT, and TT) on loss of ambulation in DMD. The authors found that the CC genotype, compared with CT/TT, was associated with earlier loss of ambulation (HR: 2.10, 95% CI: 1.45–3.04, *p* = 0.000035) in 109 patients from the Cooperative International Neuromuscular Research Group Duchenne Natural History Study (CINRG) Exome Chip cohort. Thereafter, the authors expanded the validation to pool of 660 subjects from different DMD cohorts, including 76 patients identified as non-Hispanic European race or ethnicity from the CINRG cohort, 246 from the BIO-NMD cohort, 95 patients from the Padova DMD cohort, and 243 patients from the United Dystrophinopathy Project (UDP), resulting in an overall validation cohort model with similar findings to the aforementioned (HR: 1.16, 95% CI: 1.02–1.32, *p* = 0.02).

#### Race/ethnicity and country of residence

We identified two studies reporting evidence of effects of race/ethnicity on loss of ambulation in DMD (Table 2). Specifically, in their multi-national, prospective cohort study (described above), Bello et al. [16] found that non-Hispanic patients lost ambulation at an older age compared with their Hispanic counterparts (12.4 years vs. 9.7 years, *p* = 0.003), as well as South-Asian patients (12.4 years vs. 9.7 years, *p* < 0.001). In the second study, Hufton et al. [26] estimated the mean age at loss of ambulation at 11.6 years (138.7 months) for patients of white British heritage and 9.6 years (115.2 months) for those having South Asian heritage (*p* < 0.05).

We identified one retrospective cohort study, van den Bergen et al. [47], examining differences in age at loss of ambulation among 336 European patients residing in France, Italy, the Netherlands, or the UK. Participants from the Italy and the Netherlands were found to have significantly higher risk of becoming non-ambulatory compared with patients from the UK (HR: 1.62, *p* = 0.03; and 1.83, *p* = 0.005, respectively).

#### Level of deprivation

We identified one study reporting evidence of effects of the level of family/patient deprivation (measured using the Townsend deprivation index) on loss of ambulation in DMD (Table 2). Specifically, in their retrospective cohort study of 69 UK children with DMD (mean age: 10 years), Hufton et al. [26] estimated the mean age at loss of ambulation at 10.83 years (130.0 months) among the least deprived (i.e., patients in the top 20% of the Townsend deprivation index) and at 8.54 years (102.5 months) among those most deprived (i.e., lowest 20%).

#### Other pharmacological interventions

We identified three indirect treatment comparison studies that investigated the effect of eteplirsen on loss of ambulation (Table 2). Mendell et al. [35] pooled data from a RCT (Study 201) and an open label multiple dose extension study (Study 202) involving a total of 25 US children with DMD (mean age: 9 years). The proportion ambulatory after three years was estimated at 88.3% among patients treated with eteplirsen and at 53.8% for those not treated (*p* < 0.05). In their follow-up work, Mendell et al. [36] instead compared children treated with eteplirsen (as part of Study 201/202) with matched controls sampled from the Italian Telethon and Leuven registries (multi-country, multi-centre). In this comparison, encompassing a total of 23 patients with DMD (mean age: 9 years), the proportion ambulatory after four years was estimated at 73% and 17% for those treated and not treated with eteplirsen, respectively (*p* < 0.05). Finally, Mitelman et al. [38] pooled data from Study 201/202, as well as a retrospective cohort study (US, multi-centre), and matched those treated with eteplirsen with untreated patients from CINRG DNHS matched using propensity scores (total sample: 27 patients with DMD). The median time from study baseline to loss of ambulation was estimated at 5.09 years for those treated with eteplirsen and 3.00 years for patients not treated (*p* < 0.01). The corresponding HR (eteplirsen vs. no eteplirsen treatment) was estimated at 0.119 (*p* < 0.05).

We identified two indirect treatment comparison studies reporting evidence of effects of ataluren on loss of ambulation in DMD (Table 2). Specifically, Mercuri et al. [37] examined the effectiveness of ataluren among 181 patients with DMD included in the Strategic Targeting of Registries and International Database of Excellence (STRIDE) Registry (a multi-country, multi-centre registry of patients treated with ataluren) compared with natural history controls from CINRG DNHS (described above) matched using propensity scores. The median age at loss of ambulation among patients treated with ataluren on top of standard of care was estimated at 14.5 years and for those receiving only standard of care at 11.0 years (*p* < 0.0001). The corresponding HR (ataluren vs. no ataluren treatment) was estimated at 0.28 (*p* < 0.05). In the second study, McDonald et al. [34] compared age at loss of ambulation among participants treated with ataluren as part of an open-label study (multi-country, multi-centre) and propensity score matched natural history controls from CINRG DNHS. The median age at loss of ambulation among patients treated with ataluren on top of standard of care was estimated at 15.5 years and for those receiving only standard of care at 13.3 years (*p* = 0.0006).

We identified one study reporting evidence of effects of vitamin D and coenzyme Q10 on loss of ambulation in DMD (Table 2). Specifically, in their retrospective cohort study of 1,057 patients with DMD (mean age not reported), Wang et al. [50] estimated the proportion of patients who were ambulatory (based on patient-reported data) at 12 years of age at 72% for those treated with glucocorticoids and vitamin D and at 54% for participants only receiving glucocorticoids (*p* = 0.004). Corresponding estimates for coenzyme Q10 were 74% (glucocorticoids and coenzyme Q10) and 54% (glucocorticoids) (*p* = 0.007). The corresponding HR (glucocorticoids and coenzyme Q10 vs. glucocorticoids) was estimated at 0.68 (*p* < 0.05).

#### Magnetic resonance biomarkers

We identified six studies reporting evidence of predictive effects of magnetic resonance biomarkers on loss of ambulation in DMD (Table 2). Specifically, in a prospective cohort study (ImagingDMD) encompassing 160 US patients with DMD, Barnard et al. [52] found that increased magnetic resonance spectroscopy (MRS) fat fraction (FF) of the vastus lateralis (VL) and soleus (SOL), and T_2_ in magnetic resonance imaging (MRI) of the VL, and biceps femoris long head (BFLH), all were significant predictors of loss of ambulation over 12 months (*p* < 0.001).

Godi et al. [54] estimated the accuracy for discriminating between ambulatory and non-ambulatory patients at 92% for signal intensity ratio (SIR) quadriceps, 96% for SIR flexors, 92% for thigh muscle volume index (MVI), 85% for calf MVI, 96% for biceps femoris MVI, and 100% for soleus MVI (all *p* < 0.05), in a sample of 26 Italian patients with DMD.

In an analysis of 104 patients from the ImagingDMD study (also reported by Barnard et al. [52]), Rooney et al. [56] found that the average age at half-maximal muscle involvement measures (MRS FF VL and FF SOL) were significantly associated with age at loss of ambulation (all *p* < 0.000001).

Sherlock et al. [57] report results from analyses of data recorded as part of a phase 2, randomized, placebo-controlled clinical trial evaluating the myostatin inhibitor domagrozumab among 120 patients with DMD. The authors found that total thigh MVI below median baseline value, FF of the muscle bundle, and FF of lean muscle were significant risk factors for loss of ambulation across the two-year follow-up (all *p* < 0.029).

In a prospective cohort study, Naarding et al. [55] studied 22 Dutch patients with DMD, and found that MRI FF VL was significantly associated with loss of ambulation (HR [percent-point increase]: 1.15, *p* = 0.003).

Finally, Fischmann et al. [53] reported results from a prospective cohort study of 20 Swiss patients with DMD. MRI FF of the left and right quadriceps and hamstrings, respectively, were found be significantly correlated with age at loss of ambulation (all *p* < 0.001), and the sensitivity and specificity for predicting loss of ambulation (at 50% cut-off for FF) was estimated at 100% and 91% for the left leg (*p* < 0.0001) and 100% and 100% for the right leg (*p* < 0.0001), respectively.

### Risk of bias

In total, 15 (33%) studies [18, 34–38, 40, 43, 49–54, 57] were judged to be characterized by risk of bias as assessed using the NOS (Table 2). Reasons for a risk of bias included uncertain representativeness owing to the lack of details concerning confirmation of diagnosis of DMD (*n* = 5) [40, 49–52], limited comparability owing to inadequate description of the distribution of age and/or glucocorticoid exposure (or indirect comparison of cohorts) (*n* = 9) [18, 34–38, 43, 49, 53], insufficient duration of follow-up (*n* = 4) [52–54, 57], and self-reported outcome data (*n* = 2) [49, 50].

## DISCUSSION

In this systematic review, encompassing a total of 45 publications reporting results from observational research involving children and adults with DMD from 17 countries, we synthesized the body of evidence of predictors of loss of ambulation in DMD. There is a sizeable body of evidence demonstrating that glucocorticoids prolong independent ambulation in children with DMD. Specifically, in our meta-analysis, patients treated with glucocorticoids had, on average across follow-up, more than 50% lower risk of becoming non-ambulatory compared with their untreated counterparts (overall HR: 0.44). We also found evidence pertaining to specific treatment properties, including type of agent (i.e., deflazacort vs. prednisone or prednisolone) [12, 33], regimen (i.e., daily vs. weekly vs. intermittent) [12], and duration of exposure [15, 28]. Yet, estimates of the mean and median age at loss of ambulation was found to vary across both exposed (range: 8.60–10.30 years) and non-exposed cohorts (range: 10.00–13.40 years) (Fig. 2). These findings indicate that other factors influence the trajectory of motor function and lower extremity impairment and disability in patients with DMD, as well as response to therapy.

We found evidence that age at onset of signs or symptoms of DMD can predict age at loss of ambulation. However, in line with expectations, considering that subsequent factors –in particular the disease management, including the timing of clinical interventions [4, 58] –significantly influence this milestone, the effect size was relatively modest (HR: 0.90 [22, 24]). We also found evidence that age at loss of common motor milestones (e.g., rising from the floor, climbing stairs, and running) [27] and categories of baseline 6MWT results (i.e.,<350 vs. ≥350 metres [39] and <330 vs. ≥330 metres [32]), as well categories of baseline NSAA, Gowers test, and the 10 meter timed test results [32], are associated with age at loss of ambulation in DMD. Considering the non-trivial inter- and intra-patient variability observed for these measures in previous research, further study of clinically relevant cut-offs to maximize predicted ability would be expected to help inform the design of future studies of pharmacological interventions targeting DMD.

In our systematic review, we found evidence that deletion of exons 3–7 [17, 49], proximal mutations (upstream intron 44) [31], single exon 45 deletions [49], and mutations amenable of skipping exon 8 [24, 49], exon 44 [13, 17, 24, 45, 49], and exon 53 [41], were associated with later loss of ambulation in DMD. On the other hand, distal mutations (intron 44 and downstream) [31], deletion of exons 49–50 [49], and mutations amenable of skipping exon 45 [24], and exon 51 [24, 49] were related with earlier loss of ambulation in DMD patients. The correlation between different genotypes and the likelihood of loss of ambulation impacts in the general care of patients with DMD in terms of anticipating the progression of the disease and predicting clinical function in individual single patients, as well as the accurate design and interpretation of clinical trial results involving novel therapeutic options to avoid bias resulting from unbalanced subgroups stratification. Furthermore, special caution should be considered for exon skipping trials when analyzing if the selected matched control group is appropriate to allow discrimination of outcomes given that, as it was previously mentioned, certain mutations amenable of exon skipping could potentially have a poor [24, 49] or better [13, 17, 24, 45, 49] outcome, primarily related to the specific genotype rather than the treatment.

In terms of genetic modifiers, some evidence suggests that specific single-nucleotide polymorphisms in *CD40* gene rs1883832 [18], *LTBP4* gene rs10880 [16, 23], *SPP1* gene rs28357094 [16] and rs11730582 [21], and *TCTEX1D1* genes rs3816989 and rs1060575 [43] might be associated with loss of ambulation. Some of these modifiers are modulating the expression of genes that are implicated in downstream pathways of dystrophin deficiency and regeneration; however, further investigations are required in order to better understand how these genetic modifiers impact in the natural history of DMD.

We found evidence that non-Hispanic patients and those of white British heritage on average become non-ambulatory at an older age than their Hispanic and South Asian counterparts, respectively [16, 26]. Potential reasons include differences in the distribution of specific *DMD* mutations and genetic modifiers (discussed above), as well as heterogeneity in the DMD care.

We found evidence from the UK that the least deprived patients on average become non-ambulatory markedly later than their most deprived counterparts (10.83 vs. 8.54 years) [26]. These data highlight the importance of facilitating access to timely and well-coordinated care of DMD across all socio-economic groups of society.

We found two indirect treatment comparison studies [34, 37] quantifying the benefits of ataluren –an orally administered, small-molecule compound for nonsense mutation DMD promoting readthrough of an in-frame premature stop codon to enable the production of full-length dystrophin –in terms of loss of ambulation. In both reports, patients treated with ataluren (sourced from the STRIDE Registry and a multi-country, multi-centre open-label study) were found to remain ambulatory for a significantly longer duration compared with propensity-score matched natural history controls from CINRG DNHS. However, the indirect comparison did not allow to match for all predictors of loss of ambulation as delineated in this review (e.g., genotype). Similar results have been reported as part of other observational studies of ataluren, more recently in Sweden [59].

We found three indirect treatment comparison studies [35, 36, 38] quantifying the effects of eteplirsen, a phosphorodiamidate morpholino oligomer promoting dystrophin production by restoring the translational reading frame of the *DMD* gene in patients with gene mutations amenable to exon 51 skipping. Compared with standard of care, a greater proportion of patients treated with eteplirsen remained ambulatory after three years of therapy (88.3% vs. 53.8%), as well as four years (73% vs. 17%), and a difference in the time to loss of ambulation at 2.09 years.

In regard to the usefulness of MRI, over the last decade there has been increasing interest not only in using this tool for predicting functional loss in patients with DMD but also as a possible sensitive biomarker of disease progression for clinical trials. In our systematic review, we found six studies that assessed MRI to predict clinical function [52–57]. Interestingly, most of these studies showed some evidence of either increased MRS FF or MRI T2 intensity in the vastus lateralis alone or in the whole quadriceps as significant predictor of loss of ambulation over 12 months [52–56]. In addition, involvement of the knee flexors (biceps femoris or all hamstrings) [52–54] and the soleus [52, 54, 56] were also strongly associated with the loss of ambulation in DMD. In addition, Godi et al. and Sherlock et al. measured the muscle volume index (MVI) as an independent predictor and reported a statistically significant association of the thigh MVI [54, 57], the calf, biceps femoris and soleus MVI [54] and the loss of ambulation. The application of quantitative MRI as a biomarker of the disease and as a primary surrogate outcome in clinical trials requires further investigations to better understand the progression of muscle deterioration in the images, the correlation with clinical outcomes (such as the 6-minute walk test) and to confirm these findings in larger cohorts.

As expected given the observational nature of included records, a proportion of studies (33%) were found to exhibit risk of bias. This mainly pertained to issues commonly seen in research based on real-world data of patients with DMD, such as case ascertainment (since DMD previously did not have dedicated diagnosis classification code), but also incomplete description of important confounding variables (i.e., glucocorticoid exposure), uncertain validity of outcome variables (e.g., patient self- vs. physician-reported data), and insufficient duration of follow-up (in prospective studies). Our assessment of risk of bias helps to further establish the certainty of evidence of predictors of loss of ambulation in this indication.

Our synthesis of predictors of loss of ambulation in patients with DMD have several implications for clinical practice and research. First, the results help delineate the expected trajectory of lower extremity impairment and functional disability in relation to frequently observable endogenous factors (such as DMD mutation), as well as modifiable exogenous factors (such as pharmacological therapy), which also help advise patients and families about expected clinical outcomes and the overall disease prognosis. Secondly, our portfolio of predictors will help inform patient selection criteria and sampling procedures in future research, as well as variable selection for new data collection infrastructure/disease registries and endpoint selection for clinical trials (e.g., the NSAA [60] and MRI). Thirdly, and last, our summary of predictors helps design indirect comparison studies –which are not uncommon in the field of DMD due to the limited number of cases and alternative treatments resulting in single-arm trial designs –including, but not limited to, variable selection for stratification and/or matching strategies and propensity score algorithms.

Key strengths of our work include the unrestricted search strategy. Still, it is important to keep in mind that we did not consider grey literature, or conference abstracts to allow for meaningful synthesis. As a result, evidence for some of the identified predictors may not have been fully synthesized. Additionally, since all included studies were observational in nature, conclusions regarding causality should be made with caution. It is also worth noting that our systematic review only considered evidence pertaining to patients with DMD. Given that there is no clear clinical differentiation between DMD and related dystrophinopathies, such as BMD, we therefore may have excluded studies of patients with milder phenotypes (in which cases may have erroneously been labelled as non-DMD). Finally, in the absence of evidence on loss of ambulation, some recently approved therapies were not covered in our synthesis (e.g., vamorolone [an oral, selective, dissociative glucocorticoid] [61] and delandistrogene moxeparvovec [an adeno-associated virus vector-based gene therapy] [62].

In conclusion, our review and synthesis of predictors of loss of ambulation in DMD –encompassing clinical, genetic, demographic, socio-economic, and pharmacological factors –contribute to the understanding the natural history of disease and informs the design of new trials of novel therapies targeting this heavily burdened patient population.

## Declarations

## FUNDING

This study was funded by PTC Therapeutics.

## CONFLICTS OF INTEREST

Dr Alemán reports being sub-investigator of clinical trials in DMD sponsored by Pfizer and Reveragen, and receiving a research grant from PTC. Ms Zhang, Dr Werner, and Dr Tomazos are employees of PTC Therapeutics and may own stock/options in the company. Professor Lochmüller reports being principal investigator of clinical trials in DMD sponsored by Pfizer, PTC Therapeutics, Santhera, Sarepta, and Reveragen. Professor Kirschner reports support for clinical research and/or advisory activities from Biogen, Novartis, Roche, Sarepta, Scholarrock, PTC Therapeutics, and Pfizer. The remaining authors have no conflicts of interest.

## DATA AVAILABILITY STATEMENT

The data supporting the findings of this study are available within the article and/or its supplementary material.

## SUPPLEMENTARY MATERIALS

The supplementary material is available in the electronic version of this article: https://dx.doi.org/10.3233/JND-230220.

The supplementary materials contain full bibliographic search strings as part of three tables.

eTable 1: Search terms for MEDLINE ALL (including MEDLINE daily, MEDLINE ePub ahead of print, MEDLINE In-Process)

eTable 2: Search terms for Embase

eTable 3: Search terms for the Cochrane Database of Systematic Reviews

## Supplementary Material

Supplementary Material
