# Supplementary material for: Predictors of Loss of Ambulation in Duchenne Muscular Dystrophy: A Systematic Review and Meta-Analysis
Source: J Neuromuscul Dis. 2024 Apr 30;11(3):579–612. doi: 10.3233/JND-230220 (PMC11091649; doi:10.3233/JND-230220)
Supplement: Supplementary Material [file jnd-11-jnd230220-s001.docx]

Supplemental Material

**eTable 1: Search terms for MEDLINE ALL (including MEDLINE daily, MEDLINE ePub ahead of print, MEDLINE In-Process)**

| **Step** | **Search terms** | **Hits** |
| --- | --- | --- |
| 1 | exp Muscular Dystrophy, Duchenne/ | 6,903 |
| 2 | (Duchenne and dystro*).mp. | 13,836 |
| 3 | 1 or 2 | 13,836 |
| 4 | (prognos* or (disease adj3 course) or (disease adj3 impact) or natural history or (disease adj3 predict*) or (disease adj3 outcome) or (disease adj3 progres*)).mp. | 1,452,337 |
| 5 | 3 and 4 | 1,697 |
| 6 | (comment or letter or editorial or notes or review).pt. | 5,197,988 |
| 7 | (exp animals/ or exp invertebrate/ or animal experiment/ or animal model/) and (human/) | 10,176 |
| 8 | (exp animals/ or exp invertebrate/ or animal experiment/ or animal model/) not 7 | 10,792 |
| 9 | 6 or 8 | 5,208,622 |
| 10 | 5 not 9 | 1,358 |
| 11 | Limit 10 to dt=20000101-20221231 | 1,156 |

**eTable 2: Search terms for Embase**

| **Step** | **Search terms** | **Hits** |
| --- | --- | --- |
| 1 | exp Duchenne muscular dystrophy/ | 18,848 |
| 2 | (Duchenne and dystro*).mp. | 22,469 |
| 3 | 1 or 2 | 22,469 |
| 4 | (prognos* or (disease adj3 course) or (disease adj3 impact) or natural history or (disease adj3 predict*) or (disease adj3 outcome) or (disease adj3 progres*)).mp. | 2,175,996 |
| 5 | 3 and 4 | 3,227 |
| 6 | (comment or letter or editorial or notes or review).pt. | 5,007,694 |
| 7 | (exp animal/ or exp invertebrate/ or animal experiment/ or animal model/) and (human/) | 13,152 |
| 8 | (exp animal/ or exp invertebrate/ or animal experiment/ or animal model/) not 7 | 10,053 |
| 9 | 6 or 8 | 5,017,719 |
| 10 | 5 not 9 | 2,651 |
| 11 | Limit 10 to dc=20000101-20221231 | 2,389 |

**eTable 3: Search terms for the Cochrane Database of Systematic Reviews**

| **Step** | **Search terms** | **Hits** |
| --- | --- | --- |
| 1 | [mh "Muscular Dystrophy, Duchenne"] | 4,614 |
| 2 | (Duchenne and dystro*):ti,ab,kw | 788 |
| 3 | #1 or #2 | 4,614 |
| 4 | (prognos* or (disease NEAR/3 course) or (disease NEAR/3 impact) or natural history or (disease NEAR/3 predict*) or (disease NEAR/3 outcome) or (disease NEAR/3 progres*)):ti,ab,kw | 6,214 |
| 5 | #3 and #4 | 45 |
| 6 | #5 with Publication Year from 2000 to 2022 | 45 |
